# Performance of Nanocomposites of a Phase Change Material Formed by the Dispersion of MWCNT/TiO_2_ for Thermal Energy Storage Applications

**DOI:** 10.3390/ma15093063

**Published:** 2022-04-22

**Authors:** Maha AlOtaibi, Mohammed Alsuhybani, Maha Khayyat, Bandar AlOtaibi

**Affiliations:** 1 King Abdul Aziz City for Science and Technology, Riyadh 11442, Saudi Arabia; mralotaibi@kacst.edu.sa (M.A.); mkhayyat@kacst.edu.sa (M.K.); 2 The Center of Excellence for Advanced Materials and Manufacturing, King Abdulaziz City for Science and Technology, Riyadh 11442, Saudi Arabia; bmalotaibi@kacst.edu.sa; 3 The National Center for Energy Storage Technology, King Abdulaziz City for Science and Technology, Riyadh 11442, Saudi Arabia

**Keywords:** titanium dioxide (TiO_2_), multi-walled carbon nanotubes (MWCNTs), modified paraffin wax (PW), thermal energy storage

## Abstract

Thermal energy storage technology is an important topic, as it enables renewable energy technology to be available 24/7 and under different weather conditions. Phase changing materials (PCM) are key players in thermal energy storage, being the most economic among those available with adjustable thermal properties. Paraffin wax (PW) is one of the best materials used in industrial processes to enhance thermal storage. However, the low thermal conductivity of PW prevents its thermal application. In this study, we successfully modified PW based on multi-walled carbon nanotubes (MWCNT) with different concentrations of TiO_2_—3, 5 and 7 wt.%. The morphology of PCM and its relationship with the chemical structure and stability were characterized using scanning electron microscopy (SEM), Fourier transform infrared (FTIR) and Thermogravimetric analysis (TGA). As a result, the composites achieved a highest latent heat enthalpy of 176 J/g, in addition to enhanced thermal stability after 15 thermal cycles, and reliability, with a slight change in latent heat observed when using a differential scanning calorimeter (DSC). The thermal conductivity of the composites could significantly be enhanced by 100%. Compared to pure paraffin, the PCM composites developed in this study exhibited an excellent preference for thermal energy storage and possessed low cost, high reliability, and phase change properties.

## 1. Introduction

In order to maintain current resources, achieve balance, and meet the requirements of future generations while simultaneously fostering economic development, the world’s population growth and the accompanying economic growth inevitably will be accompanied by increases in energy consumption, whether in the form of fuel, electricity, or the desalination of water. Modern industries and research organizations have focused on the use of renewable energy sources rather than oil and gas [1] The storage of thermal energy is an approach that seeks to make solar energy a profitable and effective component of the electrical grid and to maintain a balance in energy demand between seasons [2]. Thermal energy storage systems are classified into three categories, i.e., the storage of sensible heat, the storage of thermo-chemical energy, and the storage of latent heat. Regarding the sensible heat storage system, thermal energy is stored by heating or cooling a liquid or solid media, such as water, molten salt (nitrates, carbonates, and chlorides), or solids (sand, ceramics, graphite, concrete, or rock) [3]. The simple act of transferring heat to a medium to raise its temperature, stores thermal energy (charging). When the temperature is decreased, this energy might be extracted (or discharged) from the same material [4]. Chemical processes are used to store energy in thermo-chemical storage. This works on the principle of reverse chemical energy, which absorbs heat and stores it before releasing it for use later. The endothermic process “charges” the storage, which is later released by an “exothermic” material. Simplified responses can demonstrate the basic principle [5]. Latent heat storage is dependent on phase change materials (PCM) that use latent heat to store energy, such as solid-to-liquid transitions. The two main kinds of PCMs are organic and inorganic PCMs. Paraffin, fatty acids, and polymers are usually found in the organic category (ethylene glycol). Salt hydrates, salt solutions, and minerals are examples of inorganic PCMs. Inorganic PCMs have several benefits, e.g., increased storage density, higher thermal conductivity, and lower cost. However, due to several drawbacks, such as supercoiling and phase separation and their high energy storage density, they are not used extensively [6]. Organic PCM has several advantages over inorganic PCM, including a higher storage density, lower cost, and higher conductivity. Organic PCMs also have strong thermal reliability, self-core properties, corrosion resistance, variable phase change temperature, chemical stability, ease of supply, and low cost [7]. Paraffin groups are the most commonly used in PCM due to their low cost, extensive availability, high heat of fusion, corrosion resistance, and high chemical and thermal stability [8]. However, their low thermal conductivity is an inherent issue regarding using paraffin in thermal storage. Therefore, many studies have been conducted in efforts to enhance the thermal conductivity of paraffin PCMs, such as dispersing high-thermal conductivity materials in paraffin, impregnating paraffin with porous materials with high-thermal conductivity, and adding carbonaceous components. PCMs have basically enlarged paraffin/graphite composites with high-thermal conductivities and excellent heat storage capacities [9]. A new approach was developed recently that improves the thermal conductivity of PCM by using expanded vermiculite as a support material for the manufacturing of a PCM-stabilized, expanded, vermiculite/paraffin combination. Many carbon capsulation materials are used for improving the thermal conductivity of paraffin. For instance, some researchers [10] constructed a high-strength 3D graphene skeleton for the polymerization and carbonization processes, and the thermal conductivity increased several times over that of pure paraffin [10]. It was found that the thermal conductivity and the electrical conductivity of HDPE/CNT/PW-3:7 were enhanced by a factor of 2.94 and 13 orders of magnitude compared with HDPE/PW-3:7, respectively. Moreover, the HDPE/CNT/PW composite has superior thermal stability and durability. Furthermore, a recent study discussed the effect of the addition of nanocomposites of multi-walled carbon nanotubes (MWCNT) into paraffin wax (PW) TiO_2_ PCM on thermal storage performance. It was observed that the uniform distribution of the CNT layers within PW was accomplished, and some enhancements in thermal conductivity were verified [11]. TiO_2_ was recently used as an encapsulated material for PCM because of its high specific surface area, high catalytic activity, high-thermal stability, high chemical stability, fire resistance, porous structure, non-toxicity, low density, and other outstanding features [12]. Porous TiO_2_ foam is both appropriate and cost-effective for the synthesis of form-stable composite PCMs for energy storage [12] due to its characteristics mentioned above. Prabhu and his colleagues [9] conducted a study to investigate the effects of embedded PW with TiO_2_-Ag composite, and they found that the thermal properties of the composite increased by as much as 10%.

In this study, we investigated the thermal properties and the performance with regard to the thermal energy storage of PW materials for PCM composites at various concentrations of 3%, 5%, and 7%. The effect of MWCNT + TiO_2_ loadings on the thermal conductivity of PCM composites was also investigated. Furthermore, the results obtained here demonstrate large improvements in the thermal and chemical stability of PCM nanocomposites. In addition, the thermal conductivity of the PCM composites increased as the MWCNT +TiO_2_ addition increased. The latent heat and thermal cycle during 5, 10, and 15 cycles of heating and cooling showed a slight change in enthalpy. With the addition of 3% nanoparticles, the storage efficiency of the paraffin increased by 10%. These findings lead to the hypothesis that PCM-MWCNTs being embedded with TiO_2_ results in the improved thermal conductivity of the PCM nanocomposite. The results from this study are compared with those obtained in the previous investigation (in terms of enthalpy change and phase transitions), as given in Table 1, where the enhanced latent heat of melting in the current study is ΔH_m_ 176.

## 2. Materials and Methods

### 2.1. Materials

The normal paraffin of type CnH_2n+2_ belongs to a family of saturated hydrocarbons with very similar properties. Paraffins between C5 and C15 are liquids, and the rest are waxy solids. They consist mainly of straight-chain hydrocarbons that have melting temperatures that range from 23 to 67 °C. Commercial-grade paraffin wax (PW) is obtained from petroleum distillation, and it is a combination of different hydrocarbons—not a pure substance. In general, the longer the average length of a hydrocarbon chain is, the higher the melting temperature and heat of fusion will be. The paraffin wax-type phase change material (PCM), with a chain length of C21 and a melting point of 56 °C, was supplied commercially by Techno Pharmachem Co., Ltd. New Delhi, India, and it was the base material in this study. Multi-walled carbon nanotubes (MWCNTs) were obtained from SkySpring Nanomaterials, Inc., Leeds, UK (product #0553CA, Lot #0553–090916). The purity of the MWCNTs was above 95%, and they had the following parameters: a diameter of 9.5 nm, a length of 1.5 μm, and a surface area that ranged from 250 to 300 m^2^/g. The titanium dioxide (TiO_2_), Grade R902, was purchased from DuPont Co., Ltd., Dalton, GA, USA. A particle size of 0.42 µm was used to modify the paraffin. The properties of MWCNT are presented in Table 2, and the other materials are mentioned in Appendix A.

### 2.2. Synthesis of PCM Composites

The paraffin preparation included the dispersion of nanoparticles in liquid PW, due to the carbon nanotubes being nonpolar and having no affinity towards polar materials.

In this case, we use the method of preparation by adding a suitable solvent to PW at 25 °C for a shorter time period to ensure ideal solubility. First, 5.0 gm of the (PW) was added to 10 mL of pentane solvent using a 25 mL container. Second, this mixture was heated in a hot plate connected to thermocouples at 85 °C and the mixture was left to completely evaporate.

**Figure 1:**
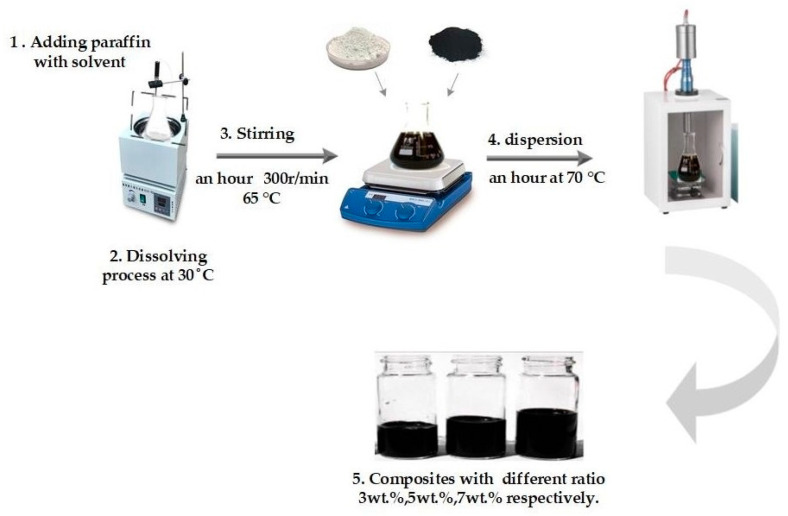
Schematic representation of the preparation methods of the composites with ratio loading nanoparticles (3 wt., 5 wt., and 7 wt.%).

To disperse the MWCNTs in PW during the heating process of PW, the MWCNTs, and TiO_2_ nanomaterials were added into the mixture gradually, followed by stirring. The rotator speed was set to 350 rpm to ensure a uniformly mixed composite, which was a black solid after cooling. In the last stage, the compound was placed in an ultrasonic bath at 80 °C for 1 h to ensure complete dispersion. Then, the samples were poured into test tubes. The concentrations of MWCNTs and TiO_2_ in PW were 3 wt.%, 5 wt.%, and 7 wt.%, respectively, as summarized in Figure 1. Table 3 presents the mass of the nanoparticles in PW used in this study.

### 2.3. Characterization

#### 2.3.1. Scanning Electron Microscope (SEM)

The morphology and microstructure of the composites (PW + MWCNTs + TiO_2_ (3, 5, and 7 wt.%) were obtained using a scanning electron microscope (SEM) (JEOL JSM-7100F, Tokyo, Japan) The samples were mounted on a flat surface after they had been coated with gold that was a few nanometers thick. The coating was exposed for two minutes to enhance its electrical conductivity and produce good-resolution images.

#### 2.3.2. Fourier Transform Infrared Spectroscopy (FTIR)

The chemical compositions of the composites were determined by Fourier transform infrared spectroscopy (FTIR) with a PerkinElmer Spectrum GX device (Hopkinton, MA, USA) that had a spectral resolution greater than 0.15 cm^−1^. The absorption ranges were from 500 to 4000 cm^−1^ at room temperature [11].

#### 2.3.3. Thermal Stability by (TGA)

Thermogravimetric analyses (TGA 1, Perkin Elmer, Shelton, CT, USA) were used to determine a material’s thermal stability, and the analyses were acquired under nitrogen at a flow rate of 20 mL/min using a thermobalance; ramp: 10 °C/min, in alumina crucibles. Each sample was heated from room temperature to 700 °C [16].

#### 2.3.4. Thermal Properties Analysis (DSC)

The phase change properties are among the distinguishing features of the compounds for latent heat storage. The latent heat of fusion and thermal cycling stability can be obtained from the differential scanning calorimetry (DSC) curves. The tests were conducted using NETZSCH, 214 Polyma for all samples at a flow rate of 20 mL/min under a nitrogen atmosphere. The temperature range of the experiment was 30 to 80 °C at a fixed heating and cooling rate of 2 °C/min.

#### 2.3.5. Thermal Conductivity

The thermal conductivity and effusively were measured at room temperature, 25 °C, using the thermal analyzer equipment C-Therm-TCi (Fredericton, NB, Canada), in which the measurements were based on the modified transient plane source method [17].

## 3. Results and Analysis

### 3.1. Morphology and Microstructure of the Composites

**Figure 2:**
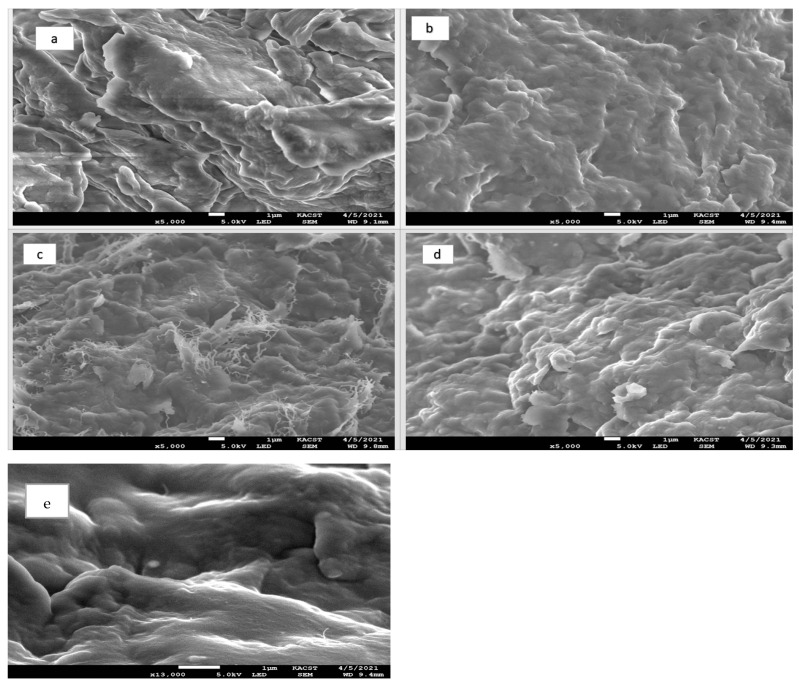
(**a**) SEM images of the pure PW and composites: (**b**) PW/MWCNT 2 wt.%; (**c**) PCM composite/T3 wt.%; (**d**) PCM composite/T5 wt.%; (**e**) PCM composite/T7 wt.%. Scale-bars of all photos are 1 µm, magnifications (up to ×5000), voltages (5.0 Kv).

The surface morphology was obtained using a scanning electron microscope (SEM). The morphology of the pure paraffin sample is shown in Figure 2a, and the paraffin composition sample with the nanotubes is displayed in Figure 2b. Finally, the composites we prepared in this study after adding TiO_2_ by various concentrations, is displayed in Figure 2c–e (details of SEM images of MWCNT shown in the supplementary materials Figure S1).

To compare them with pure PW, see Figure 2a and the SEM image of the PW/MWCNT in Figure 2b.

The crystallized micro-platelets are formed randomly with a streamlined internal structure, as it is characteristic and typical of alkanes with linear molecular structures and long carbon chains [18].

Figure 2b demonstrates the SEM images of the PW/MWCNT. As before, the paraffin wax, if crystallized, forms micro-platelets with a lamellar internal structure. A good dispersion of the nanotubes in the paraffin matrix can be observed, as the MWCNT are uniformly distributed within the sample. Areas with considerable higher content of MWCNT can be clearly observed in composite in addition. When adding TiO_2_, as shown in Figure 2c–e, it can be observed that a layer of nanoparticles uniformly covered the surface of the PW/MWCNT, thus, an even distribution of particles occurred. This demonstrates that there was a slight increase in the average particle size due to the increase in the nanoparticles’ mass ratio with no evidence of nanotubes aggregation or entanglement, which indicated an ideal dispersion of nanoparticles on paraffin sample.

### 3.2. Chemical Composition

**Figure 3:**
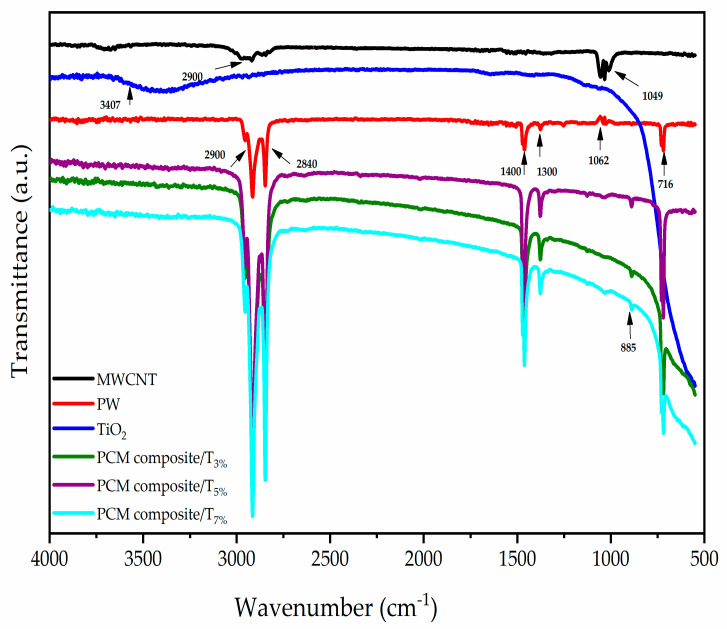
FTIR spectra of composites.

The chemical compositions of the composites were determined by Fourier transform infrared spectroscopy (FTIR) analysis. Figure 3 demonstrates that most of the absorption ranges of PCM composites were in the range of 2900–2800, and two absorption peaks were shown at 2900–2840 cm^−1^; they correspond to the expansion oscillations between CH_2_. This means that there is an alkene group. Additionally, there is a wide absorption range between 1400 and 1300 cm^−1^ that can be attributed to CH2.

In addition, the disappearance of the absorption spectra on MWCNT at 1049 and on PW at 1062, is attributed to the formation of a new spectral band at 820, which was attributable to a C–H bend (meta). The spectrum of TiO_2_ shows an absorption broadband in the region of 800−400 cm^−1^. In addition, the spectra for the MWCNT displayed a band of absorption around 3000 cm^−1^, which can be accredited to the stretching of the C–C bonds. Stretching vibrations were also observed between C and C at around 1000 cm^−1^. The disappearance of the spectrum in the range of 1034–1006 was attributable to the formation of N–H. The functional group in all modified samples is CH–CH_2_–NH_2_. Similar absorption spectra were obtained for all the samples [19]. In addition, as the intensity of the characteristic absorption peak of the PCM composites was unchanged with the loading of nanoparticles, there was no significant band shift compared between concentrations. Which indicated that the MWCNT/TiO_2_ composite structures were synthesized successfully. 

### 3.3. Evaluation Stability of PCM Composites

**Figure 4:**
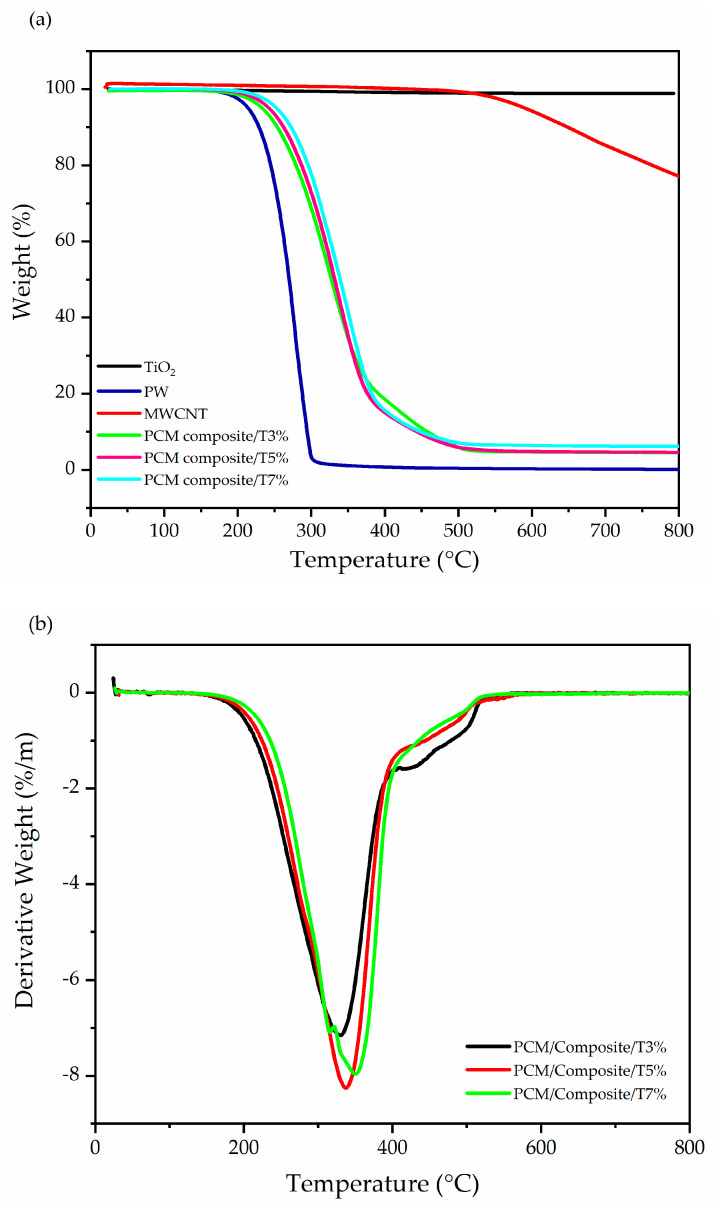
(**a**) Thermogravimetric analyzer (TGA) plot of the composites (**b**) derivative thermogravimetry curves of PCM composites.

Thermogravimetric analyses were used to determine a material’s thermal stability, as shown in Figure 4. The TGA curves of PW and composites correspond to 3.0, 5.0 and 7.0 wt.%. It was evident that at 210 °C, pure PW starts the weight degradation, while maximum deterioration occurred at approximately 300–470 °C, and no residue remained at 600 °C. For the nanocomposite samples, the onset of deterioration occurred at temperatures 253, 262 and 270 °C, respectively, for the concentrations 3, 5, and 7 wt.%, and a weight loss in the range of 4.7–6.5 wt.% occurred. These results indicate that the behavior of the samples is a typical one-step thermal degradation. They also show rapid weight loss with almost no residual charcoal remaining in the 600 °C temperature range for PW due to chain degradation [20]. In addition, the MWCNT is stable up to 535 °C, and TiO_2_ particles are very strongly thermally stable until 800 °C, without the general loss of mass on the TGA curve, and the chemical stability often ensures the long life of materials. This indicates that the added excellent thermal stability of pure PW means that the nanoparticles enhance the physical bonding between PW molecules due to their low densities, and the weight loss that occurred as evidenced in the thermal behavior curves is shown in Table 4. This confirms that the CNT/ TiO_2_ is well-dispersed in the PW according to the TGA analysis mentioned in our previous publication [11].

### 3.4. Thermal Property Measurement

**Figure 5:**
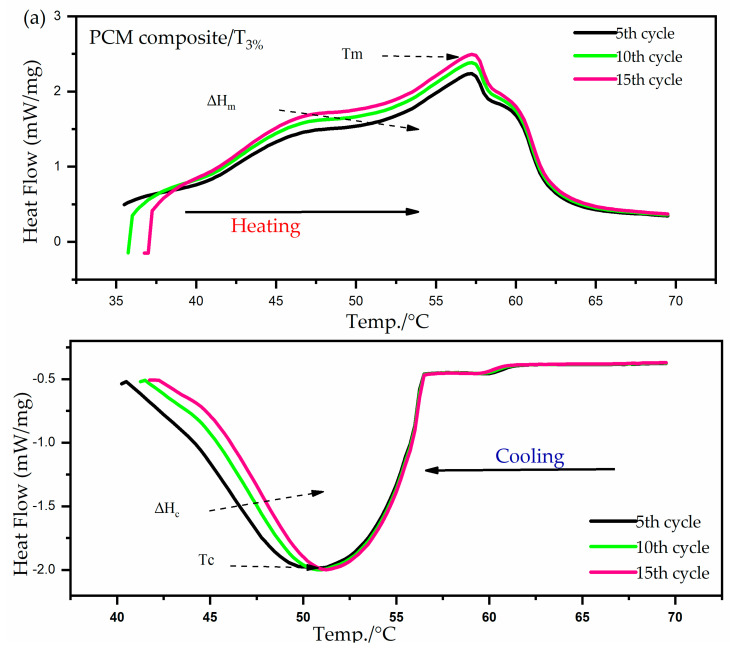

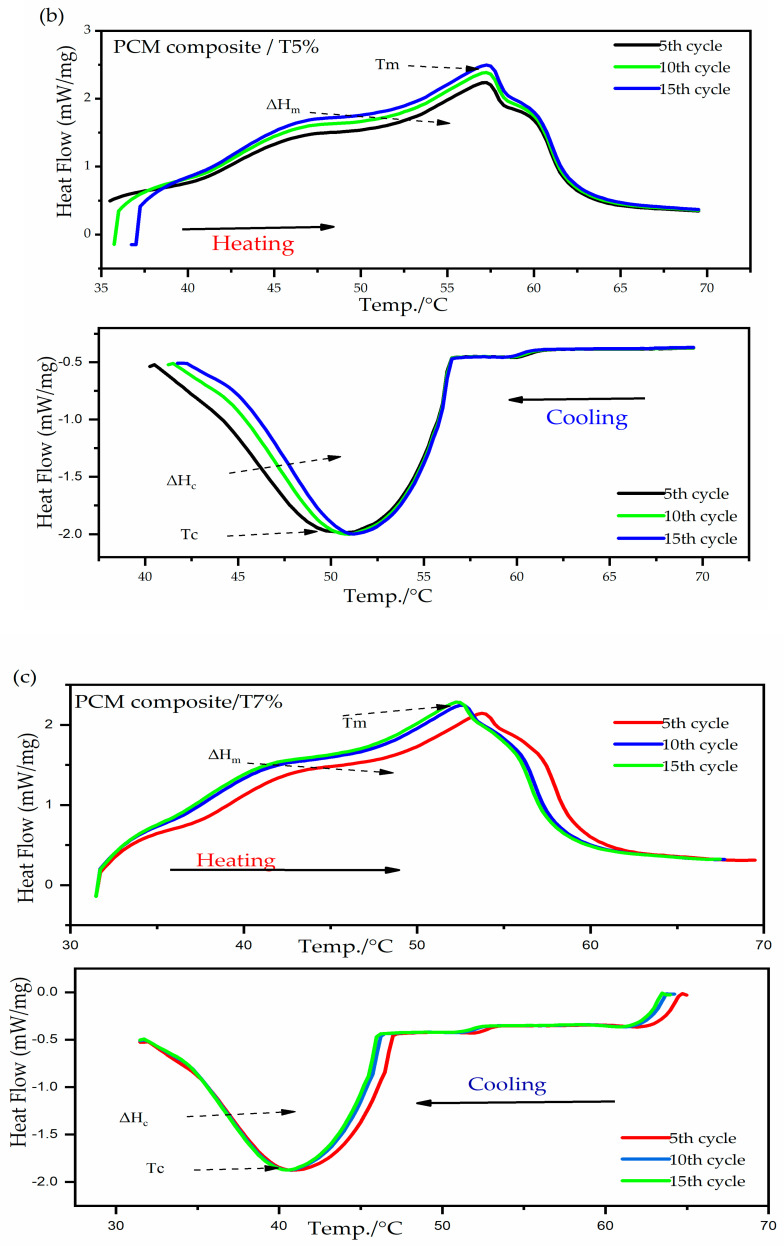

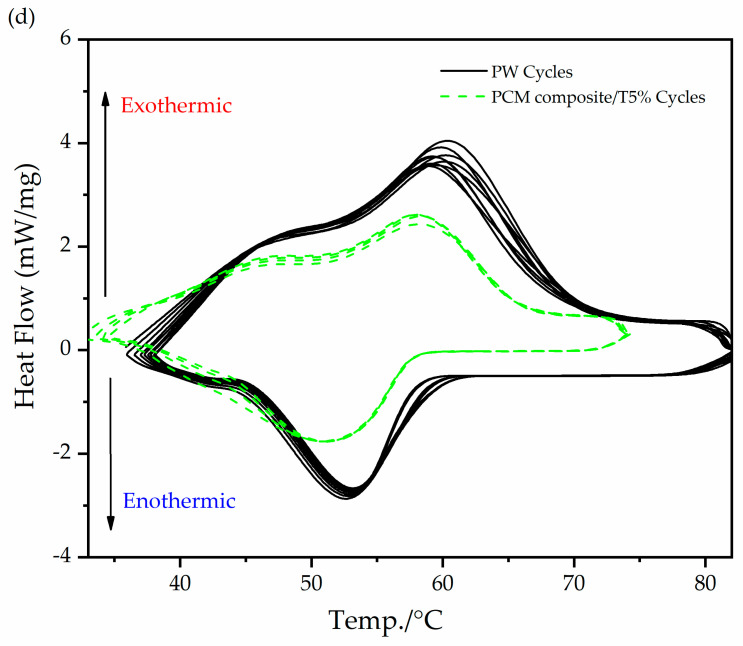
(**a**) DSC thermograms and cycle of a PCM composite 3%. (**b**) PCM composite 5% and cycle. (**c**) PCM composite 7% and cycle. (**d**) Comparing composite 5wt.% with paraffin after different cycles.

The characteristic feature of a PCM is the phase change from liquid to solid and vice versa. This makes it a good thermal energy storage material. The melting and freezing processes were given as the melting (Tm) and freezing temperatures (Tf), respectively. The (Tm) and (Tf) of pure PW were 35–70 °C and 58–45 °C, respectively [13], and the results are shown in Figure 5 (details of DSC curves of PCM composites and their recycling shown in the supplementary materials Figure S2), while the data are summarized in Table 5. In a comparison of the melting/freezing point temperatures with the samples modified by nanoparticles at 3, 5, and 7 wt.%, respectively, the melting and freezing points were in the range of 34–64.2 °C and 56–31 °C, respectively. The enthalpy of the phase transition was calculated between the melting point/cooling point temperatures, and Table 4 shows that the melting enthalpy and the freezing enthalpy of pure PW were 221.31 and 112.67 J/g, respectively. In comparison with the samples modified with 3% and 5% nanoparticles, the freezing enthalpies were 176.2, 150, 134, 82.83, 93.5, and 78.8 J/g, so obviously the melting/freezing enthalpy of the composites decreased in comparison to pure PW, and the rate of the decrease in latent heat was slight in T3% and T5%.

The essential reductions in the latent heat that were observed in T7% were 87 and 43 J/g, and this means increasing the concentration of CNT. TiO_2_ substantially reduced the enthalpy of the latent heat capacity of the composite. Therefore, the thermal conductivities of the additives were higher than the thermal conductivity of PW. According to the calculations of thermal conductivity and the temperature difference experiment, additives with a higher thermal conductivity than that of PW can enhance the thermal conductivity of the PW and accelerate the phase change process [21]. Thus, the appropriate concentration should be determined in order to provide good thermal conductivity and a higher phase change enthalpy [18,19].

**Figure 6:**
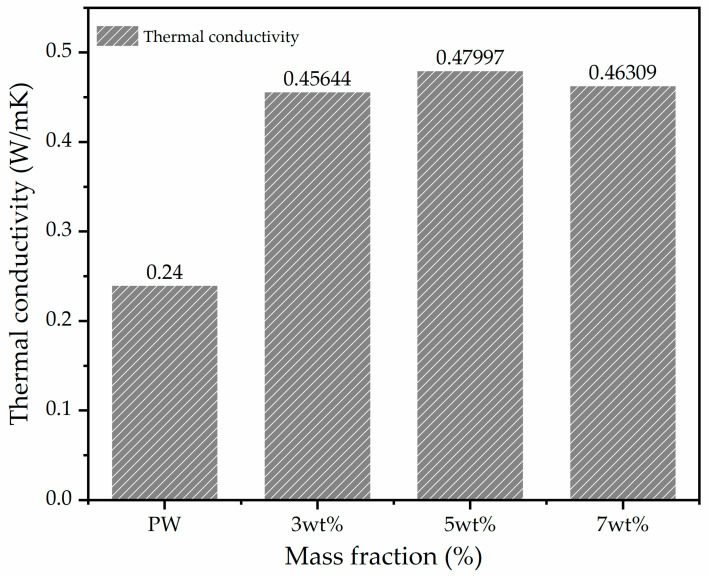
Thermal conductivity of PCM composites and PW.

Therefore, the thermal conductivity and effusively were measured at room temperature, 25 °C. As is well-known, PW has poor heat conductivity and photon transport suppression because of its lower density, which could delay the heat response to the thermal storage or release [13,22]. Thus, developing composites based on MWCNT and transition metal TiO_2_ which have thermal conductivity is important to enhance the performance of PW. Figure 6 shows the thermal conductivity of the PCM composites prepared with different concentrations of MWCNTs/TiO_2_ compared with PW. The thermal conductivity of the pure PW is 0.24 W/m, and that of the composites is 0.45, 0.47, and 0.45 W/m for concentrations of 3, 5 and 7 wt.%, respectively. We observed that the thermal conductivity of the PCM composites increases while increasing the mass fraction compared to pure PW. However, at 7 wt.%, it did not increase, maybe due to a clot of carbon nanoparticles, because the agglomeration of nanoparticles minimizes the thermal properties in the composite, and the enthalpy decreases, as shown in Figure 5. Therefore, the main challenge in this project is enhancing the thermal conductivity by preserving the high latent heat of the PCM composites. The results show that the 5 wt.% dose is the most appropriate for this purpose in this study.

We also provide a measure of the workability of the long-term use of the thermal energy system, presented in Figure 5d, for a concentration of 5 wt.%. The curves, after the first cycle, were synchronized with the fifteenth cycle, which we noticed was similar behavior to the PW, with only a slight change of less than 15 °C. This means that adding nanoparticles to PW at a concentration of 5 wt.% gives excellent results for the enhancement of thermal conductivity and high reliability after cycles.

### 3.5. Charge, Storage, and Discharge Performance

**Figure 7:**
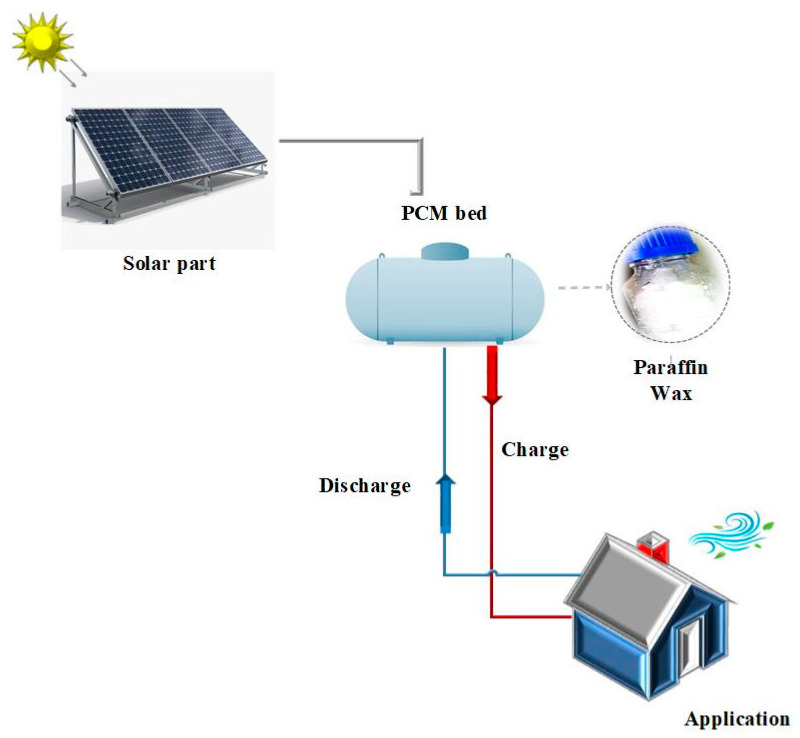
Design of conventional tank storage system in a concentrating solar system used in this study.

A thermal energy storage system consists of a cylindrical tank filled with PW connected to the solar part. An electrical heater is used to supply heat to the thermocouples, and they are located in the direction of the cylindrical tank to record the temperature of the PCM [13]. The system is controlled using a proportional integral derivative (PID) controller in order to take advantage of stored energy, which can be used at a later time for domestic or industrial applications, as seen in Figure 7.

Assisted by hot water produced in solar collectors, the thermal storage unit is filled by the PW, which changes from the solid phase into the liquid phase during charging by the hot water produced in the solar collectors. As we know, the latent heat is collected for use in phase transformation. During the night mode of operation, the hot water loop from AD undergoes a change in the configuration of the valves (i.e., the hot water loop from the solar collectors is closed, and the hot water loop between AD and the thermal storage unit is opened). The hot water loop rejects the latent heat of the changing phase (liquid to solid) from the PW, and in that way, it is heated to the temperature required for the proper operation of the desorption phase in AD.

As demonstrated in Figure 7, during the daytime, thermal storage is used to change the phase from solid to liquid, and then, during the night, the energy is rejected, adding water to be heated. During this process, liquid material PW rejects some of its heat to the water, thereby changing its phase from liquid to solid.

**Figure 8:**
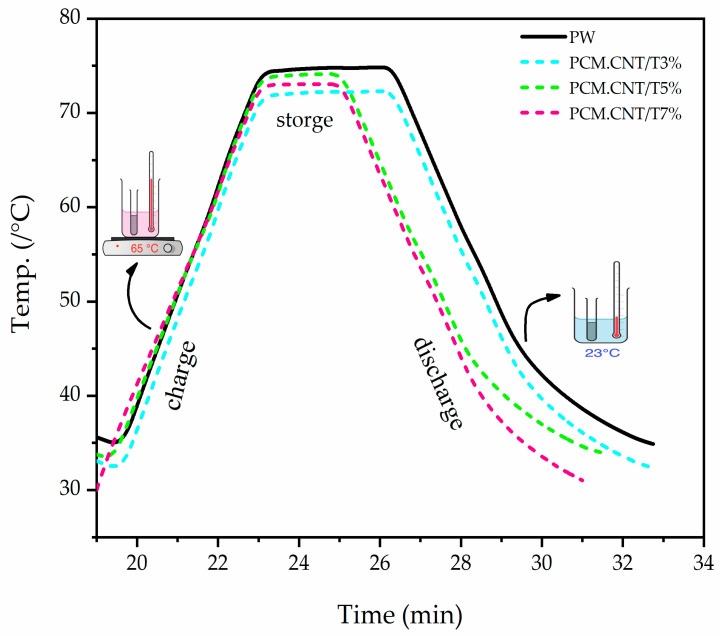
Time–temperature plot curves of latent heat storage and release storage for PW and composites.

During the charging period, upon heating, the heat is transferred to the PCM material, and the process of thermal charging begins slowly until it is completely melted. As we can observe in Figure 8, the PW and composite melting processes occur at 74, 71, 73.4, and 72.5 °C, respectively. The phase transition does not happen directly according to temperature, but instead, it occurs over a confirmed range of temperatures, which is called the phase transition range.

The experimental results illustrate the transition phases driving energy storage in pure PW and the composite 3 wt.% beginning at 15 and 12 min, respectively. We note that the storage period is similar to the storage period of PW because the loading of nanocomposites is low. Meanwhile, the period given for the PCM composites 3 and 7 wt.% are 10 and 9 min, respectively, and the storage efficiency of the pure PW and composites is 87% 91%, 97%, and 95%, respectively. This time decrease in the thermal storage corresponds to the increase in the heat transfer due to the enhancement of the thermal properties due to the addition of CNT/TiO_2_.

When the temperature decreases, the samples begin to solidify and release heat—this period is called the discharging period. It was also observed that the time required for full latent heat to be released clearly decreased when we added more of the additives. This may be ascribed to a smaller time scale for melting and cooling phase transitions caused by a faster scanning rate. To improve this situation [23], the heating and cooling rates should be chosen with longer periods to store and release heat.

The PCM composite that contained 5 wt.% of TiO_2_ exhibited a slower rate of decrease in the temperature among the three produced composites. 

## 4. Conclusions

Thermal energy storage technologies are considered enabling technologies for energy production technologies, such as solar thermal, and concentrating solar power. PW was used in this study as a PCM with nanocomposites, including MWCNTs and TiO_2_ at concentrations of 3, 5, and 7 wt.%, to enhance the thermal properties of the base material of PW. The following conclusive remarks can be drawn from the current detailed investigations:

- Considering the textural properties of the modified 5 wt.% CNT/TiO_2_ ratio, they have a great influence on the microstructure and thermal properties of the resultant nanocomposites, and the dispersion, composition, and structure, and thermal stability were studied by SEM and TGA, respectively. The SEM micrographs of the various composites showed no tendencies to segregate. The chemical composition of the composite PCM was verified by FTIR.
- Thermal conductivity analysis shows that the dispersion effect of nanoparticles is the basic starting point for the TC improvement of the PCMs. This indicates that the addition of nanoparticles resulted in a good performance in PCMs.
- It was observed that the melting and recrystallization curves corresponded well for each sample with the highest latent heat as compared to previous studies.
- The thermal reliability and durability of the nanocomposites were evaluated by DSC with scans for the 15th cycles of heating—cooling cycles for a concentration of 5 wt.%. This was found to be consistent with the PW cycles.
- Based on the measurements of enthalpy, the results confirm that the nanocomposites developed in this work have great potential for the applications of cycles in commercial-grade PW, showing high fusion heats [22], and are safe and non-reactive. They are compatible with all-metal containers, and they can easily be incorporated into heat storage systems. Commercial PW, which melts at around 55 °C and has a latent heat of melting at about 210 kJ/kg, has been used by a large number of investigators. When “selecting a PCM for a particular application, the operating temperature of the heating or cooling should be matched to the transition temperature”; thus, the PCM that is selected should have a melting temperature in the range of 40–60 °C.

## Figures and Tables

**Table 1:** Comparison of the latent heat of the composite PCMs prepared in this study with some reported PCMs for thermal energy storage applications.

References	PCM + Additives	Fraction of Additives	Thermal Conductivity	Temperature Change Phase (Tm–Tc)	Latent Heat Enthalpy (ΔHm–ΔHc) (J/g)
[9]	Paraffin/TiO_2_ eAg þ SDS	1:0.25	0.451	59.8–61.31	124.7–139.9
[12]	Paraffin/porous TiO_2_ foam	1.5	1.059	49.27–50.65	97.27–99.05
[13]	Paraffin/expanded vermiculite (implanted with carbon)	--	0.452	48.85–53.0	101.14–103
[14]	paraffin/f-MWCNTs	3:1	0.51	48.2–54.6	119.6–122.1
[15]	TiO_2_/paraffin/SSL	19:1	0.195	60.64–60.59	165.1–167
In this study	Paraffin/MWCNT + TiO_2_	3:5:7	0.48	58–46.5	176.2–93

**Table 2:** Thermophysical properties of MWCNTs used in this study.

MWCNT	Description
Average Diameter	9.5 nm
Purity	>95 wt.%
Surface Area	250–300 m^2^/g
Amorphous carbon	SSA: >60 m^2^
Electrical conductivity	>100 s/cm
Thermal Conductivity	3000 W/m K
Density	~2.1 g/cm^3^
Manufacturing method:	Catalytic CVD

**Table 3:** Mass of nanoparticles in paraffin used in this study.

Sample Code	Paraffin, wt.%	Partials, wt.%	Total Loading, wt.%
PW	100	0 wt.%	100
PCM composite/T3%	97	2% MWCNT + 1%TiO_2_	3
PCM composite/T5%	95	2% MWCNT + 3%TiO_2_	5
PCM composite/T7%	93	2% MWCNT + 5%TiO_2_	7

**Table 4:** TGA data of the PW and various composites.

Sample Code	MWCNT/TiO_2_ Loading (wt.%)	Onset Temperature °C	Weight Loss at 600 °C
PW	100	243	0.29
CNT	100	535	94.2
TiO_2_	100	stable	99.7
PCM composite/T3%	3	253	4.7
PCM composite/T5%	5	262	4.8
PCM composite/T7%	7	270	6.4

**Table 5:** Melting and cooling points provided by PW and composites and thermal cycling properties of melting latent heat (ΔHm) and cooling latent heat (ΔHc).

Sample	Melting Process			Cooling Process		
	Phase Change Temperature Tm (°C)	ΔHm (J/g)		Phase Change Temperature Tc (°C)	ΔHc (J/g)	
		First Cycle	15th Cycle		First Cycle	15th Cycle
Pw	60	221.2	210.5	53	−112.2	−109.6
PCM composite/T3%	57	176.2	173.3	42.4	−82.83	−80.47
PCM composite/T5%	58.2	150.1	150	46.5	−93.5	−92.0
PCM composite/T7%	58.25	134.9	134.7	49.5	−78.8	−76.28

## Data Availability

Data sharing is not applicable to this article.

## Supplementary Materials

The following are available online at https://www.mdpi.com/article/10.3390/ma15093063/s1, Figure S1: SEM of MWCNT. Figure S2: (1) DSC curve of PCM composites; (2) recycling of PCM composites.

## Appendix A. Properties of Material We Used in This Study from the Company

**Table A1:** Thermal properties of paraffin and used nanoparticles.

Item	Index
MWNTs	95%, 30–50 nm
Purity	>95 wt.%
Outside diameter:	30–50 nm
Length:	5–20 um
Amorphous carbon	SSA: >60 m^2^
Electrical conductivity	>100 s/cm
Bulk density	0.28 g/cm^3^
True density:	~2.1 g/cm^3^
Manufacturing method:	Catalytic CVD

**Table A2:** Thermal properties of titanium dioxide R-902+.

Item	Index
TiO_2_ content,% (m/m)	≥93
Carbon Black Undertone	10.0–14.0
Hegman Fineness	7 min
Hegman End Point	4 min
Hegman Scattered Particles	15 max
Alkyd Gloss @ 20 °C	60 min
Oil Absorption (g/100 g)	13.0–20.0
pH	7.3–9.5
Resistance @ 30 °C (kOhmem)	4.00
Appearance	White powder
Thermal conductivity	4.8 W/m·K
Average dimeter	0.42 µm

**Table A3:** Thermal properties of paraffin wax (PW).

Item	Index
PW content, % (m/m)	90–100%
Physical and Chemical Properties	Wax-like prills/flakes/cubes/slabs.
Odor	Odorless
Melting Point	56C (133F)
Thermal conductivity	0.3 W/m·K
