# Supplementary material for: Performance of Nanocomposites of a Phase Change Material Formed by the Dispersion of MWCNT/TiO2 for Thermal Energy Storage Applications
Source: Materials (Basel). 2022 Apr 22;15(9):3063. doi: 10.3390/ma15093063 (PMC9100833; doi:10.3390/ma15093063)
Supplement: Supplementary file 1 [file materials-15-03063-s001.zip › materials-1571040-supplementary.pdf]

materials

Supplementary Materials

Performance of Nanocomposites of a Phase Change Material Formed by the Dispersion of MWCNT/TiO_2 for Thermal Energy Storage Applications

Maha AlOtaibi [1], Mohammed Alsuhybani [1,*], Maha Khayyat [1] and Bandar AlOtaibi [2,3]

[1] King Abdul Aziz City for Science and Technology, Riyadh 11442, Saudi Arabia; mralotaibi@kacst.edu.sa (M.A.); mkhayyat@kacst.edu.sa (M.K.)
[2] The Center of Excellence for Advanced Materials and Manufacturing, King Abdulaziz City for Science and Technology, Riyadh 11442, Saudi Arabia; bmalotaibi@kacst.edu.sa
[3] The National Center for Energy Storage Technology, King Abdulaziz City for Science and Technology, Riyadh 11442, Saudi Arabia
* Correspondence: sohybani@kacst.edu.sa

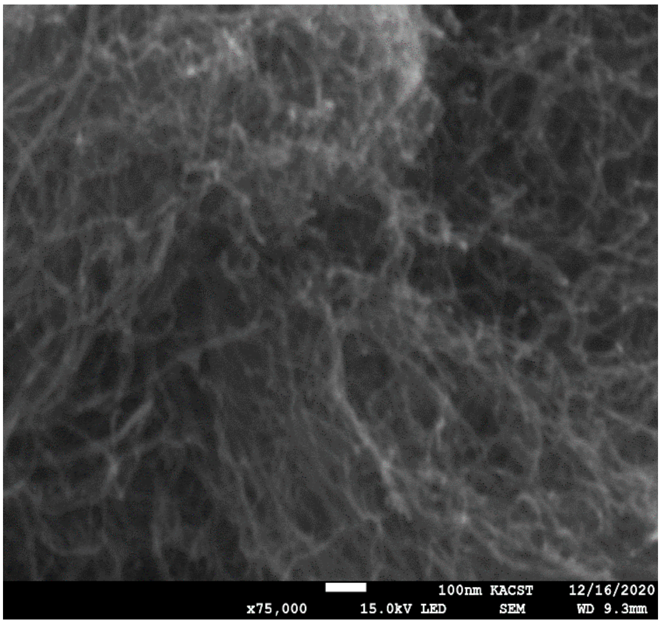

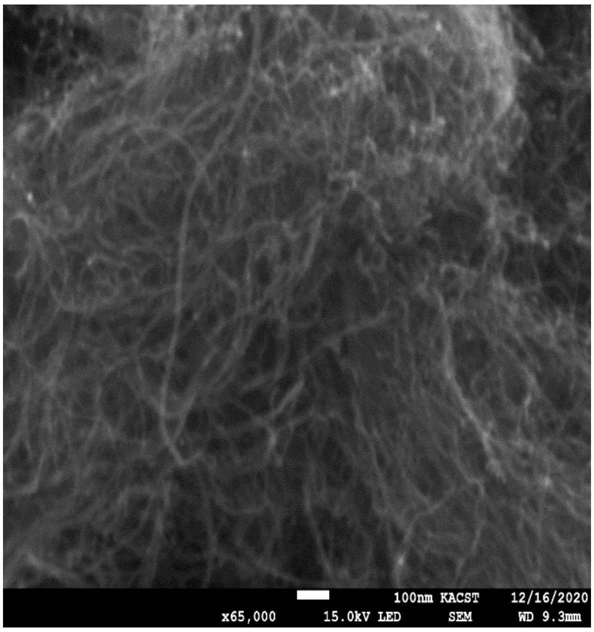

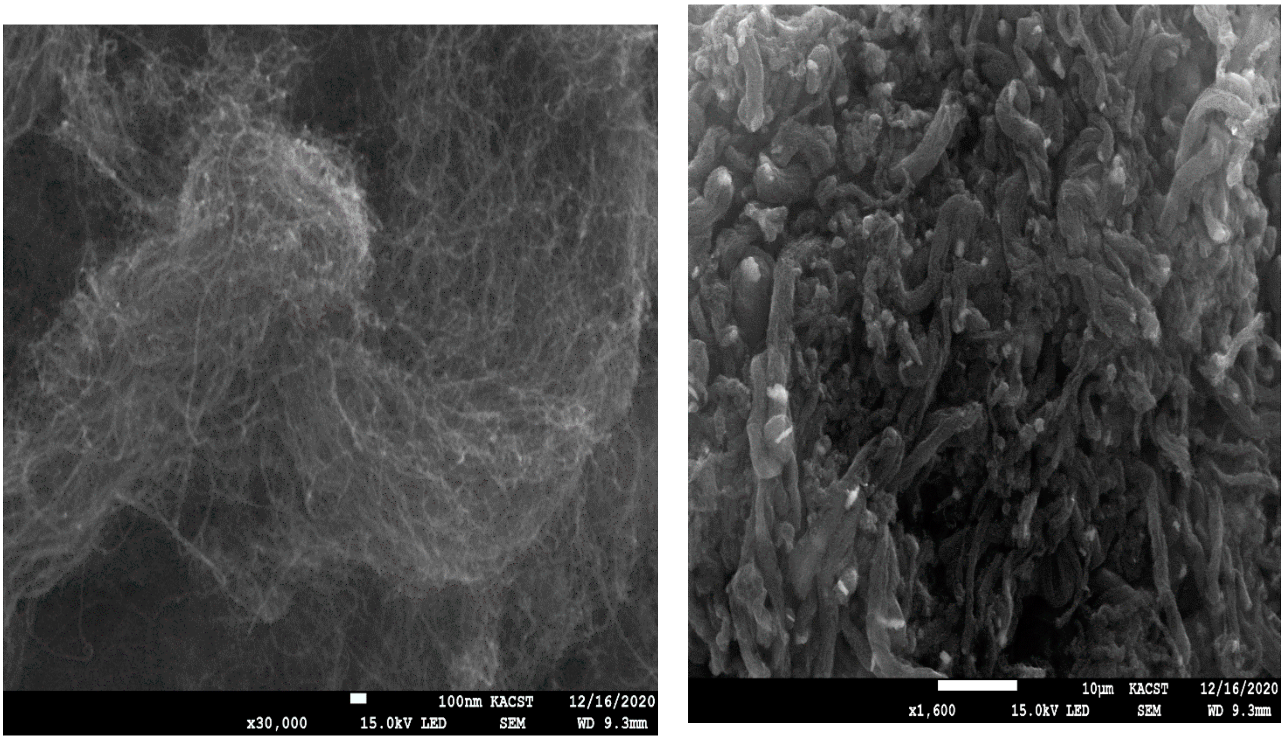

Figure S1. SEM of MWCNT.

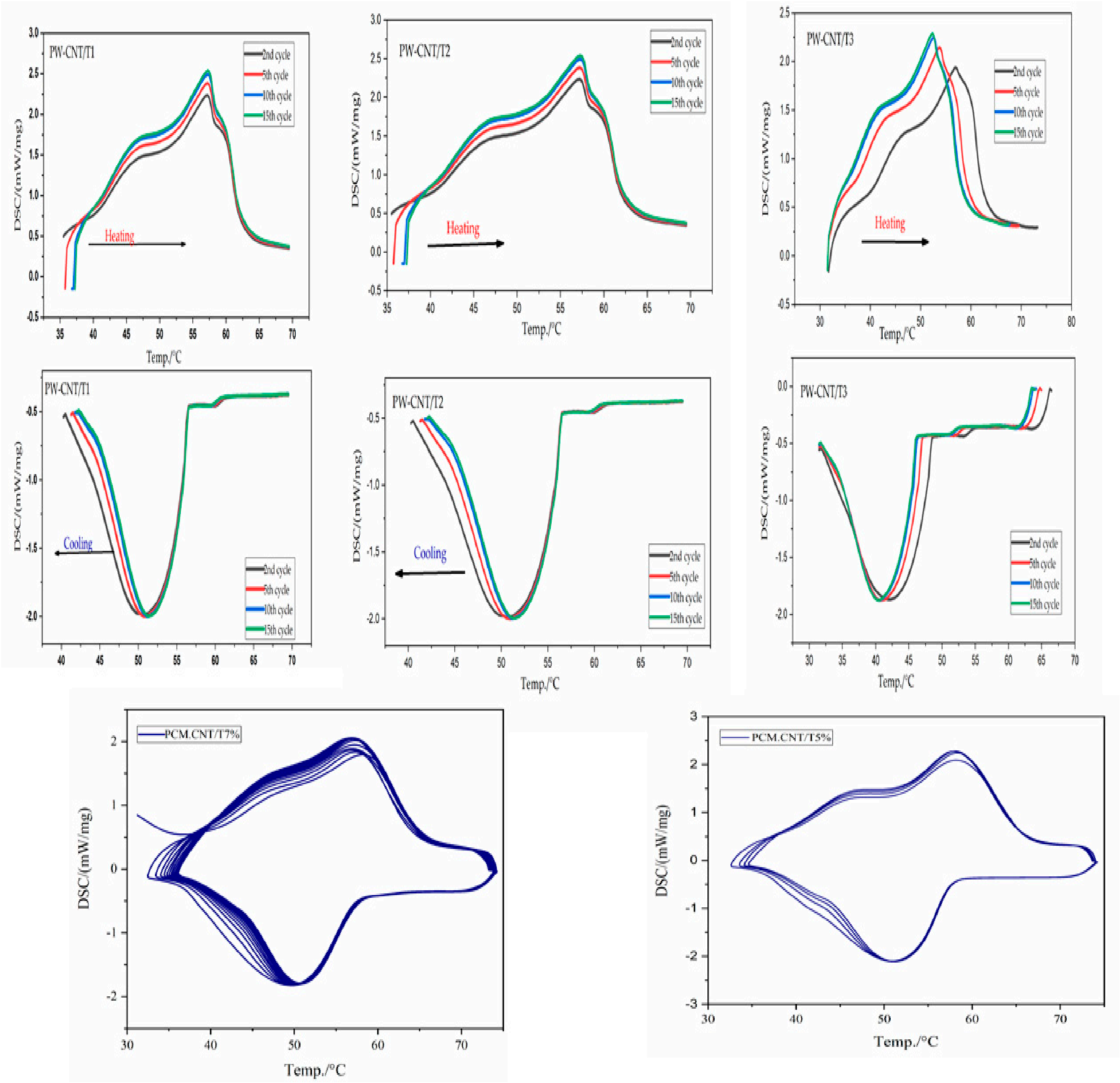

Figure S2. 1) DSC curve of PCM composites; 2) recycling of PCM composites.
